# Guiding crowds when facing limited compliance: Simulating strategies

**DOI:** 10.1371/journal.pone.0276229

**Published:** 2022-11-11

**Authors:** Christina Maria Mayr, Gerta Köster

**Affiliations:** 1 Department of Computer Science and Mathematics, Munich University of Applied Sciences HM, Munich, Germany; 2 Department of Informatics, Technical University of Munich, Garching, Germany; University of Shanghai for Science and Technology, CHINA

## Abstract

At traffic hubs, it is important to avoid congestion of pedestrian streams to ensure safety and a good level of service. This presents a challenge, since distributing crowds on different routes is much more difficult than opening valves to, for example, regulate fluid flow. Humans may or may not comply with re-directions suggested to them typically with the help of signage, loudspeakers, apps, or by staff. This remains true, even if they perceive and understand the suggestions. Yet, simulation studies so far have neglected the influence of compliance. In view of this, we complement a state-of-the-art model of crowd motion and crowd behavior, so that we can vary the compliance rate. We consider an abstracted scenario that is inspired by a metro station in the city of Munich, where traffic regulators wish to make some passengers abandon the obviously shortest route so that the flow evens out. We investigate the effect of compliance for two very simple guiding strategies. In the first strategy, we alternate routes. In the second strategy, we recommend the path with the lowest crowd density. We observe that, in both cases, it suffices to reroute a small fraction of the crowd to reduce travel times. But we also find that taking densities into account is much more efficient when facing low compliance rates.

## Introduction

### Background and overarching goal

Major events cause crowds to gather not only at the event location but also at traffic hubs. For example, the traffic volume on public transport increases significantly before and after soccer matches [1, 2]. As a result, many subway stations experience congestion [3]. In extreme situations, such as the Munich Oktoberfest or the Tokyo rush hour, trained crowd managers alleviate the situation. Self-sufficient guidance systems may help when no human crowd manager can be on site.

Several concepts for guiding crowds have been proposed [4–12] but, to our knowledge, none of these have been realized yet. A possible explanation for this could be that, so far, there is no system that meets the many, often conflicting, requirements: As many people as possible should be transported; the level of service should be high and safety guaranteed; at the same time, the system must cope with the fact that crowds are heterogeneous and never behave the same way; moreover, implementation and maintenance should be cheap and simple while, ideally, the system should adapt to low and high traffic volumes. But mostly, we believe, that no system addresses the fact that compliance is imperfect. Not all people perceive or understand the instructions, and those who do may choose not to follow.

Accordingly, the overarching goal of this study is to better understand the effect of imperfect compliance on attempts to regulate pedestrian flows. Since we want to address a diverse readership from engineering, computer science, and crowd management, we need to specify some terminology, before we can fully describe the research gap and formulate suitable research questions.

### Terminology

Looking at the various systems and concepts that have been developed to guide people, we find that studies present similar ideas without citing each other, e.g. [4, 5], and that they use terms differently. We attribute this to the wide variety of disciplines that traffic researchers come from. To avoid further confusion, we explain how we understand important terms in this contribution in Table 1.

**Table 1:** Meaning of terms in this study.

Term	Meaning
Compliance	Following an instruction, usually a route suggestion, irrespective of whether a person has already decided to choose a specific route.
Compliance rate	Percentage of people who follow instructions.
Guiding strategy	Algorithm that dynamically generates measures to guide a crowd. The algorithm may do this, e.g., by comparing measurements of quantities of interest, such as flow rates, to desired values.
Measure	Action derived from the guiding strategy. E.g. giving instructions that people should take a certain exit.
Medium	Medium that is used to transport the information about the measure, e.g. dynamic lights.

We also propose to distinguish between guiding individuals and guiding crowds. Guiding individuals implies furnishing information that enables them to improve their personal situation without regard to the whole crowd, or even at the cost of others. For this, so-called Advanced Traveller Information Systems (ATIS) “acquire, analyze, and present travel information to individuals” [13]. One expects an individual to comply if he or she believes that this is advantageous. ATIS are common for motorists, for example Trafficmaster [14] or Vehicle Information Communication System [15]. Sato et al. [12] propose an ATIS for pedestrians. Here, we are interested in guiding crowds, which is often referred to as crowd management or pedestrian traffic management [16]. The goal is to make an event or an evacuation safer for all by distributing, stopping, or redirecting flows of people. One expects compliance to improve when people share a social identity that will make them support actions that help the group [17–19].

We define a guiding strategy as an algorithm that dynamically generates measures to guide a crowd. The measures are put in place through instructions. Such strategies usually come from crowd management and pedestrian flow control. We do not consider static measures like the planning of facilities [16, 20], placing obstacles [21], or static evacuation plans [6], as guiding strategies. If the algorithm relies on measurements, sensors must be put in place, e.g. video surveillance [22], or smartphones receiving and transmitting signals [23–25]. According to our definition, guiding strategies correspond to the tactical or strategic layer described in [26], that is, we do not look at so-called ‘control models’ [27] that operate at the locomotion layer. We would like to stress, that one should not confuse the guiding strategy, that is, the algorithm that generates measures, with the measures or medium used to implement it. The medium transports information necessary to carry out the measures. Media can be static or dynamic signs, barriers, loudspeaker announcements, instructions in an app, auxiliary equipment like robots [28], background music [29] or crowd managers on site [30].

Instead of the concept of guiding strategy, several studies [4, 5, 9, 10, 31] use the term controller. We find this misleading in the context of crowds because, in control theory, when one talks of controllers, one assumes a system that one can indeed fully control. But crowds do not always behave the same way, people may fail to perceive or understand instructions or even reject them, making full control rather elusive. In addition, the term controller itself is ambiguous. For example, Hoogendoorn et al. [32] denote the individuals themselves as controllers.

It is important to keep in mind that the success of a guiding strategy depends on whether or not people follow instructions. This is usually captured by the term compliance. Yet, we have not found an explicit definition.

We understand compliance as following an instruction, here a route suggestion, irrespective of whether a person has already decided to choose a specific route [33]. In other words, it does not necessarily imply a change of mind.

For a population, we define the compliance rate as the proportion of people who accept recommendations by the guidance system. Thus, we distinguish between full and partial compliance. We expect full compliance when people have no choice but to accept a measure, e.g. when barriers are put in place. We expect partial compliance when people merely receive information or recommendations, e.g. through lights, or apps or crowd managers. Partial compliance makes developing guiding strategies much more challenging. We need to take peoples’ reactions into account because, otherwise, we risk to overestimating the guiding strategy’s performance. In this contribution we investigate the effect of varying compliance rates on two simple and, as we argue, easy-to-implement guiding strategies.

### Crowd management: State-of-the-art

#### Experimental studies on the effect of information provision methods

To our knowledge, no true guiding strategy has been implemented so far in experimental setups, let alone in life traffic. There are, however, a vast number of studies on the effect of how information is provided. For a survey, we refer to [34]. One interesting study is in [35] where the authors examine how arrows stimulate people’s path choice. They even alter the arrows’ direction every three minutes, which could be interpreted as the kernel of an alternating route algorithm. However, their focus is on the effect of the information provision so they do not study any strategic aspects.

#### Guiding strategies and guiding algorithms

Guiding strategies or algorithms do not always target route choice: Molyneaux et al. [7], for instance, dynamically place obstacles to separate counterflows in their simulation. Zhang et al. [22] propose a model that adjusts the walking speed of pedestrians. In the models proposed by [10, 11, 31], the pedestrians’ free-flow velocities are adjusted. The free flow speeds are the speeds at which pedestrians would walk when the path is free. It remains unclear how walking speeds or free-flow velocities can actually be adjusted in real systems.

However, the majority of guiding strategies try to redirect people. That means, they influence their route choice. All of the following studies use computer simulations. In [5], the authors propose so-called on-off-controllers that open and close routes. They do not discuss the reaction of a crowd facing shutting routes. Ren et al. [4] simulatively investigate the performance of on-off controllers and so-called Proportional Integral controllers. These rely on influencing the attractiveness of exits by so-called evacuation assistants, that change their movement, or the sound or light they emit. The aim is to make the density in front of a particular exit correspond to a desired density. PI controllers consistently calculate an error value as the difference between a desired and a measured process variable, then apply a proportional (P) and integral (I) correction. We fear, that the volatile compliance of a crowd may seriously degrade such an intricate system. Lopez-Carmona et al. [8] intend to make the egress of a soccer stadium safer. They divide the stadium into cells and dynamically recommend exits depending on the cell a person is in. To find an optimal strategy, they use a tabu search algorithm that minimizes a new density-based safety criterion. Their optimization strategy is very complex and relies on a non-standard metric. Guo [36] and Li et al. [37] use dynamic signage to minimize evacuation times. They assume that every pedestrian perfectly follows instructions. Alqurashi et al. [38] describe an agent-based traffic simulator for cars and pedestrians. The goal is to offer an environment to test guiding strategies. Gao et al. [39] switch signals on and off in the hope to reduce densities and travel times in a simulated evacuation scenario. They also investigate what effect partially available or delayed measurement data have within their model.

#### Modeling route choice

Route choice models describe a person’s route decision based on environmental and exogenous factors [8]. Numerous experiments have been conducted to identify such factors [23, 40–55]. Typical are route lengths and expected travel times. For more background reading, we refer to [34].

Also, several route choice models have been proposed and studied in simulations. Daamen [56] considers walking and waiting times, route lengths and walking comfort. Xu et al. [5] suggest a model that takes the distance to the exits and their attractiveness into account. Ramos et al. [33] propose logit models to investigate how travel time affects route choice. Arganda et al. [57] model the route choice of animals. Tong et al. [48] use this model for humans and extend it by allowing individuals to become less sensitive to environmental information the more decisions they have to make. Tong et al. [49] introduce a ‘route commitment effect’ that says that the more people invest into a planned route by walking further along it, the bigger their tendency to stick to this route. Aleksandrov [58] put forward a logit model to describe the route choice between lifts and stairs in tall buildings. Haghani et al. [59] calibrate a model that considers the distance to exits, congestion at the exits, the flow towards the exits, and the visibility of the exits. Haghani et al. [60] suggest another logit model to capture people’s tendency to maintain an initial exit choice. Lopez [8] consider the distance to the target, corridor widths, congestion at different locations, the number of previous route changes, and the effect of information. For this, they split the population into agents who follow the route recommendation with a probability that corresponds to the compliance rate and others that decide according to the route choice model.

Many route choice models implicitly address compliance, e.g. by introducing ‘inertia’ [60]. However, with the exception of Lopez et al. [8], they do not vary compliance, thus, neglecting uncertainty in the simulation outcomes.

To date, there is no generally accepted model for route choice behavior that can be relied on to develop guiding strategies. We attribute this to the multitude of factors, the influence of which has not been clarified so far. For example, it is still not understood under which circumstances people follow others [61]. In addition, there is the dependency of the route choice on the way information is provided, such as loudspeaker announcements or lights. In particular, we find that the reaction to recommendations is rarely modeled or that the model has not been validated. Finally, complex guiding strategies are often tailored for one specific use case and cannot be easily generalized. All this may explain why, to our knowledge, none of these systems have been realized in practice.

### Research questions

We argue that it depends on the route choice behavior, and in particular on the compliance rate how well a guiding strategy performs. In view of this, we find that the complexity of many guiding strategies ill fits the accuracy of the reaction model that the modelers assume. Indeed, the assumption often is full compliance, which we cannot agree with. This motivates our research questions:

- To what degree do simple heuristics suffice to guide people?
- What is the effect if only a portion of the people complies with the route recommendation?

### Use case and specified research question

Our research questions are inspired by a real-life use case. At the metro station Münchner Freiheit in Munich, soccer fans take the underground train to get to the stadium. This causes congestion in certain areas, in particular, along the shortest, and preferred, route. As a consequence, the travel times and the density are high, and the level of service is low. In the hope to improve the situation, Munich’s traffic managers are looking for ways to make just the right amount of passengers abandon the shortest path in favor of two alternative options.

Accordingly, we set ourselves the goal of shortening the travel times and reducing congestion by redirecting new arrivals to alternative routes. We will describe how we abstract the very complicated set-up at the station in section *Modeling the scenario: simplification of the topography*.

We would like to emphasize that, in this study, we do not assume any specific way to provide route information. We imagine that it could be, for example, in the form of a dynamic arrow pointing to the entrance of the currently recommended corridor.

We also want the complexity of the strategy to fit the rest of the model where all aspects, ranging from human behavior, to the rendering of the environment, to measurements of crowd densities and travel times are strongly simplified. We consider a high degree of abstraction to be inevitable since the whole system is subject to many pronounced uncertainties.

In the same spirit, we opt for two guiding strategies that are based on simple heuristics: In the first, we alternate routes. The second strategy recommends the corridor where the density is currently lowest. With this we specify our research questions and rephrase them as one:

- How well do the heuristic guiding strategies perform regarding density and travel times if we do not know the compliance rate?

### Structure of the study

The rest of the study is organized as follows. In section *Materials and methods*, we briefly introduce our software tools. In section *Design of the study* we describe the scenario and in *Simulation model* the two guiding strategies that we investigate. The outcome of the computer experiments, in particular the effect of compliance, follows in *Simulation results*. In the *Discussion* we summarize the results and reflect limitations. Finally, we address follow-up research in the *Conclusion*.

## Materials and methods

For our computer experiments, we build on existing software, namely the *Vadere* simulation framework for pedestrian dynamics. *Vadere* offers a variety of locomotion models of which we choose the well-validated *Optimal Steps Model*. In the *Optimal Steps Model* pedestrians make steps that bring them closer to their destination while skirting obstacles and avoiding collisions with others. In this, the *Optimal Steps Model* is a classic model of pedestrian dynamics. For details, we refer to [62].

**Fig 1:**
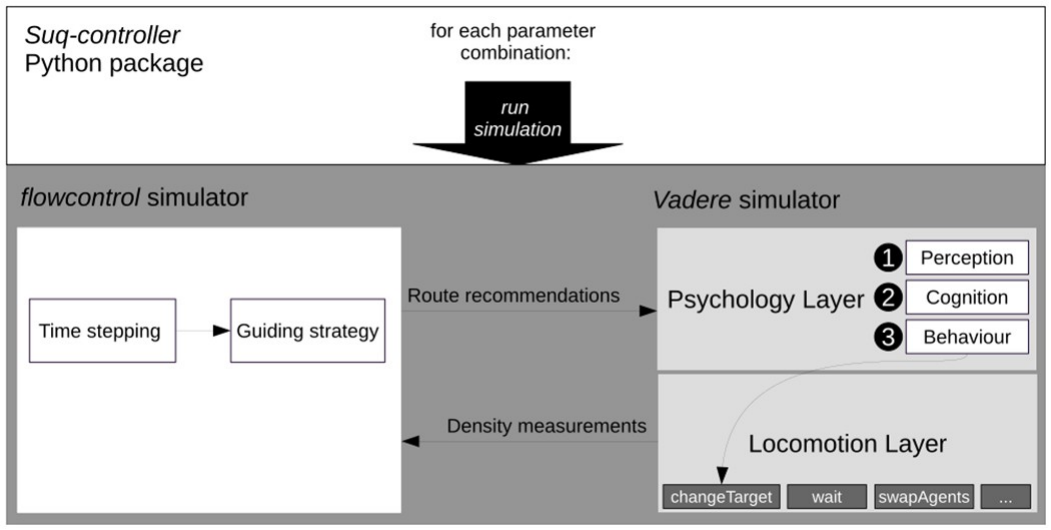
Overview of simulators and their models. The *suq-controller* selects a parameter sample and triggers the simulation. *flowcontrol* steps ahead in time (10 seconds in this study), to call a guiding strategy. The strategy passes route recommendations to the *Vadere* simulator, where agents perceive and process the information, possibly adapt their behavior by selecting another target destination, and walk towards their destinations. *Vadere* sends density measurements back to *flowcontrol* for consideration.

We implement behavioral changes as reaction to guidance in the psychology layer of *Vadere*. The guiding strategies themselves are implemented in a Python package, *flowcontrol*, that communicates as client with *Vadere* as server in a client-server model, see Fig 1. We carry out parameter studies using the Python package *suq-controller* from the *Vadere* eco-system. All methods and models can be found in the publicly accessible repository *crownet* https://crownet.org and through www.vadere.org. All software packages and the crownet repository have been thoroughly tested according to the principle of continuous integration as described in [63].

## Design of the study

### Scenario selection

**Fig 2:**
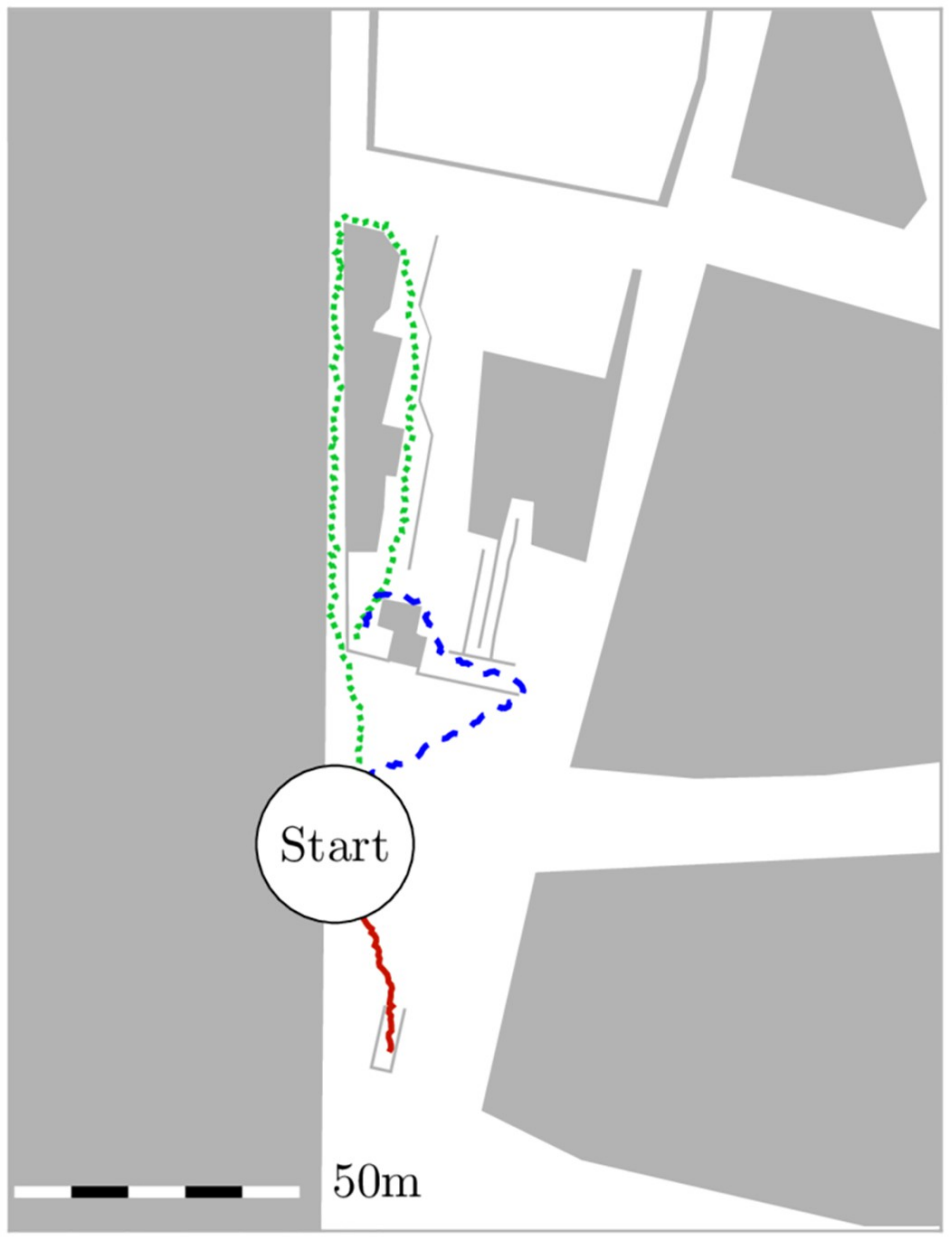
Surface level of the underground train station Münchner Freiheit in Munich. The snapshot from the *Vadere* simulator’s graphical user interface abstracts an image from Open Street Map. There are three main routes to get to the trains. The vast majority of people use the shortest route (solid line).

**Fig 3:**
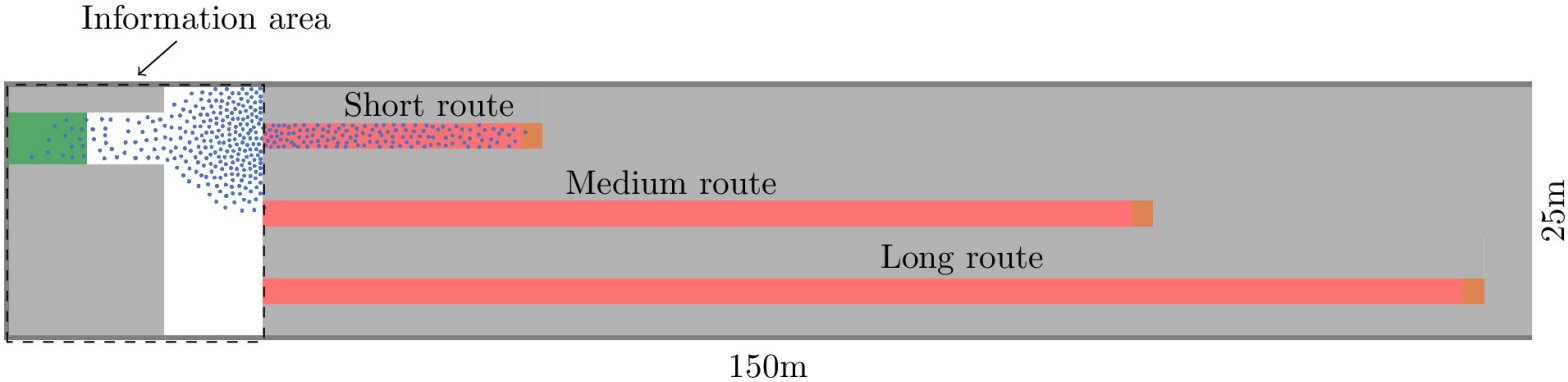
Simplified topography. Agents are spawned in the source (green) and walk to the targets (orange) on the right end of each corridor. Without guidance, all agents take the short route. In the setting with guidance, agents receive a route recommendation in the information area. They follow the route recommendation with a probability *c*, that is, the compliance rate.

Our inspiration comes from a real-life problem that arises with every major soccer game at Munich’s metro station Münchner Freiheit. We assume people experience congestion if up to 100 fans per minute arrive at the station to take the underground train to the stadium. In the real scenario, pedestrians can choose among five paths, of which two are virtually ignored. The remaining three differ mainly in length, but also in the number of twists and turns, stairs, and surface condition. See Fig 2 for a bird’s eye view. With so many influencing factors it would be difficult to separate cause from effect. Thus, since we wish to focus on the effect of varying compliance, we decided to strongly abstract the scenario to three corridors of different lengths, see Fig 3. Note that the pedestrian model’s navigation algorithm makes the agents take the shortest path by default, which perfectly fits the real behavior of local soccer fans who are familiar with their surroundings.

### Model of the dynamic pedestrian management system

According to [7], a dynamic pedestrian management system consists of two components: a guiding strategy and the crowd. Guiding strategies are defined by their objectives and by the measures taken to achieve the objectives. We aim at reducing travel times and densities as in [39]. As measure we provide pedestrians in a certain zone with information on which route they should take, following [8]. At this point, we are satisfied if we significantly improve performance, leaving optimization to a stage when the system is better understood.

Our crowd model comprises an operational and a tactical layer as suggested by [26]. The operational layer refers to pedestrian locomotion for which we use *Vadere*’s agent-based *Optimal Steps Model*, a classic model of pedestrian dynamics. On the tactical layer, we model people’s behavioral changes when they react to route recommendations. Kleinmeier et al. [64] propose three sequential phases for this: people perceive environmental stimuli, which they process in a cognition phase. Then, they react by selecting a behaviour from a behavioural repertoire. All three phases are implemented in the psychology layer of the *Vadere* simulator [64], see Fig 1. In our case, the environmental stimuli are route recommendations that are generated dynamically by the guiding algorithm. Agents in the information zone perceive the environmental stimuli. In the cognition phase, agents decide whether they follow the recommendation, which we model stochastically with a probability that corresponds to the compliance rate *c*. Compliant agents change their route when the recommendation differs from their former selection, and thus, might alter their walking direction. Non-compliant agents continue to their destinations.

### Parameter selection and quantities of interest

Our goal is to investigate the performance of the two heuristic guiding strategies that we propose for crowds with limited compliance. For this, we vary the compliance rate for each strategy. In the absence of more information, we assume it to be uniformly distributed. We observe densities, walking velocities, and individual travel times as quantities of interest.

Every 0.4*s*, we measure the density and the velocity in each corridor. See red measurement areas in Fig 3. The density is defined as [65]: *d* = *N*/*A* where *N* is the number of pedestrians and *A* is the area of the measurement section. As velocity, we use the spatial mean velocity which is defined as the average of the pedestrians’ individual velocities in the measurement area [65]. The travel time per agent is the time it takes to reach the destination. In our evaluations, we look at the median travel time as in [7].

The *Vadere* simulator collects data every 0.4*s*: 12500 density and velocity values as well as the individual travel times of 14613 agents to compute mean values, the standard deviations (std), and other statistics for the quantities of interest. The dependent and independent variables are listed in Table 2.

**Table 2:** Parameters and variables for both guiding strategies. We conduct two studies: one for the fixed order strategy and one for the minimal density strategy. The variables and parameters are identical for the fixed order and the minimal density strategy. We vary the compliance rate and measure the travel time, the density, and the velocity. We fix the only parameter of the guiding strategies, the time interval between recommendation updates, to 10*s*. We set the parameters of the locomotion model to established defaults, see supporting information (S3 Appendix).

Type	Name	Unit	Values, levels	Comment
Fixed parameter	Time interval	s	10.0	Parameter of the guiding strategies: time in between two route recommendations
Independent variable	Compliance rate	1	0.00,0.025, …., 0.975, 1.00	Value 0.00 corresponds to the setting without guidance
Dependent variable	Travel time	*s*		
	Density	*ped*/*m*^2^		
	Velocity	*m*/*s*		

With such a complex and interdependent model, there are several further model parameters that might have an influence on the results, namely the parameters of the locomotion model and of the guiding strategy. It is impossible to vary all of them at a time. We feel confident to fix the parameters of the locomotion model, the *Optimal Steps Model*, to default values which have proven to be suitable in a range of validation tests [63]. For the reader’s convenience, we state them in the supporting information (S3 Appendix). The guiding strategy’s only parameter is the time that elapses before route recommendations are updated. We fix this time interval to 10*s*, a choice that we explain in detail in the following section.

## Simulation model

### Guiding strategies based on heuristics

In view of the model’s and, indeed, the true system’s many uncertainties, we feel that simple approaches have a better chance to be implemented in a real setup than more intricate strategies. We also expect strategies to be more robust if they do not depend on precise measurements to achieve target values in some control loop. Thus, we propose two guiding strategies based on simple heuristics:

- The ‘fixed order strategy’ sequentially alternates route recommendations every Δ*t* = 10*s* for a topography with *n* routes that all lead to the same target. After route *n*, the strategy re-starts with the first corridor. Thus, the order is fixed. In our scenario, with *n* = 3, the algorithm provides the following recommendations: *t* = 0*s*: short corridor, *t* = 10*s*: medium corridor, *t* = 20*s*: long corridor, *t* = 30*s*: short corridor, …
- The ‘minimal density strategy’ measures the density every *Δt* = 10*s* and recommends the route where the density is minimal.

Both strategies assume that information on the suggested route is provided to the virtual pedestrians in a zone in front of the scenario’s diverging corridors, see Fig 3. We imagine it to correspond to the place where soccer fans get off the bus at the surface level of the real metro station. While we do not investigate the medium used to provide the information, we envision that routes could be numbered and a dynamic sign could be placed in the information area showing the current route number or point to the corresponding path. Alternatively, the current route recommendation could be communicated using an app.

The time interval Δ*t* = 10*s* is a compromise. In an ideal world, where agents would react immediately and reliably, one would expect a smaller Δ*t* to improve the distribution of agents. Real reactions, however, are not ideal. Imagine, e.g., a dynamic arrow that switches direction every second. People will miss the information, get confused and probably annoyed. On the other hand, pedestrians walk about 10*m* in 10*s*. We find it plausible, that one re-checks direction after covering that distance.

One could come up with the idea of addressing individual agents in different directions to dispense with the timing issue. Again this does not work with humans: Groups would have to split up, which they will not. Also, people are aware of others and might be confused if suggestions differ.

The minimal density strategy does not suffer from that drawback, since one can assume that the corridor with the minimal density stays the same for several seconds. Thus, the strategy can be implemented without a time schedule. This gives us a great deal of freedom in the realization of the strategy. E.g. Schuhbäck et al. [66] devised a method to measure densities by transmitting information between mobile phones. One could use this in a mobile phone application that adds route recommendations. We would also like to mention that the minimal density heuristic can also be represented by a system of on-off-controllers as in [4, 5], see supporting information (S1 Appendix).

### Modeling the scenario: Simplification of the topography

To abstract from the real metro scenario and from Fig 2, we neglect surface conditions, obstacles and twists and turns, that could cause bottlenecks. Instead, we measure the length of the routes to create a simplified topography that represents the corridor widths and the ratio of the route lengths, see Fig 3. Stripping detail from the topography allows us to concentrate on the influence of compliance. It also saves computation time in a study where we need to generate many samples through costly simulations. The outer dimensions of the area are 150*m*x25*m*. We choose a corridor width of *w* = 2.5*m* for all corridors. Every two seconds, we spawn eight agents in a so-called ‘source’, which is the green area on the left in Fig 3. The agents try to reach the (orange) targets placed at the ends of each corridor on the right. Without guidance, all agents take the shortest route, that is, the corridor on top, which corresponds to what most soccer fans do in the real world scenario. In the setting with guidance, agents receive a route recommendation every Δ*t* = 10*s* in the information area, which is composed of the green area of the source and the white area between the source and corridors, see Fig 3. If agents linger in the information area and receive multiple recommendations, they respond only to the first recommendation. They follow the route recommendation with a probability *c*, that is, the compliance rate.

### A plausibility check for the minimum compliance rate

Schadschneider et al. [67] and Zhang et al. [65] have developed plausible formulae when to expect jamming: It occurs if the inflow *J*_*in*_ into a bottleneck is larger than the capacity *J*_*C*_ which is defined as the maximal flow in the bottleneck. Conveniently, *J*_*C*_ can be taken from measured fundamental diagrams that show the flow in dependency of the density. It must be understood as an upper limit, because jams have been observed for smaller flow values, possibly caused by flow fluctuations, effects based on local organization, or psychological aspects [68].

We want to exploit this ‘jamming criterion’ to estimate the minimum compliance rate. We ask: How many people must be redirected to alternative routes to resolve the congestion in the short corridor? We will later compare simulation results to the estimate as a plausibility check. Thinking of *J*_*C*_ as upper limit, we can estimate the lower limit *l* of the percentage of people we need to reroute.

$$\begin{array}{c} l=\frac{J_{in}-J_{C}}{J_{in}} \end{array}$$ (1)

With an inflow of *J*_*in*_ = 1.60*ped*/(*ms*) and a capacity of *J*_*C*_ = 1.30*ped*/(*ms*), we expect that we have to redirect at least 19% of the agents to resolve the congestion in the short corridor. See supporting information (S2 Appendix) for the parameter choices.

Based on this, we calculate the minimum compliance rate necessary to avoid a jam. The fixed order strategy recommends the shortest corridor to 1/3 of the people. A proportion of *q* = 2/3 of the people are directed away from it. With a compliance rate *c*, the flow in the short corridor is:

$$\begin{array}{c} J_{short}=J_{in}\left(1-cq\left(c\right)\right) \end{array}$$ (2)

To avoid a jam, we require *J*_*short*_ ≤ *J*_*C*_. Thus, the minimal compliance rate is

$$\begin{array}{c} c\geq \frac{1}{q(c)}(1-\frac{J_{C}}{J_{in}}) \end{array}$$ (3)

Plugging *q* = 2/3 into to Eq 3 yields a compliance rate of at least 28% to avoid congestion. For the minimal density strategy, *q* must be obtained from the simulation output before we repeat the calculations.

## Simulation results

### Congested routes cause high travel times

**Fig 4:**
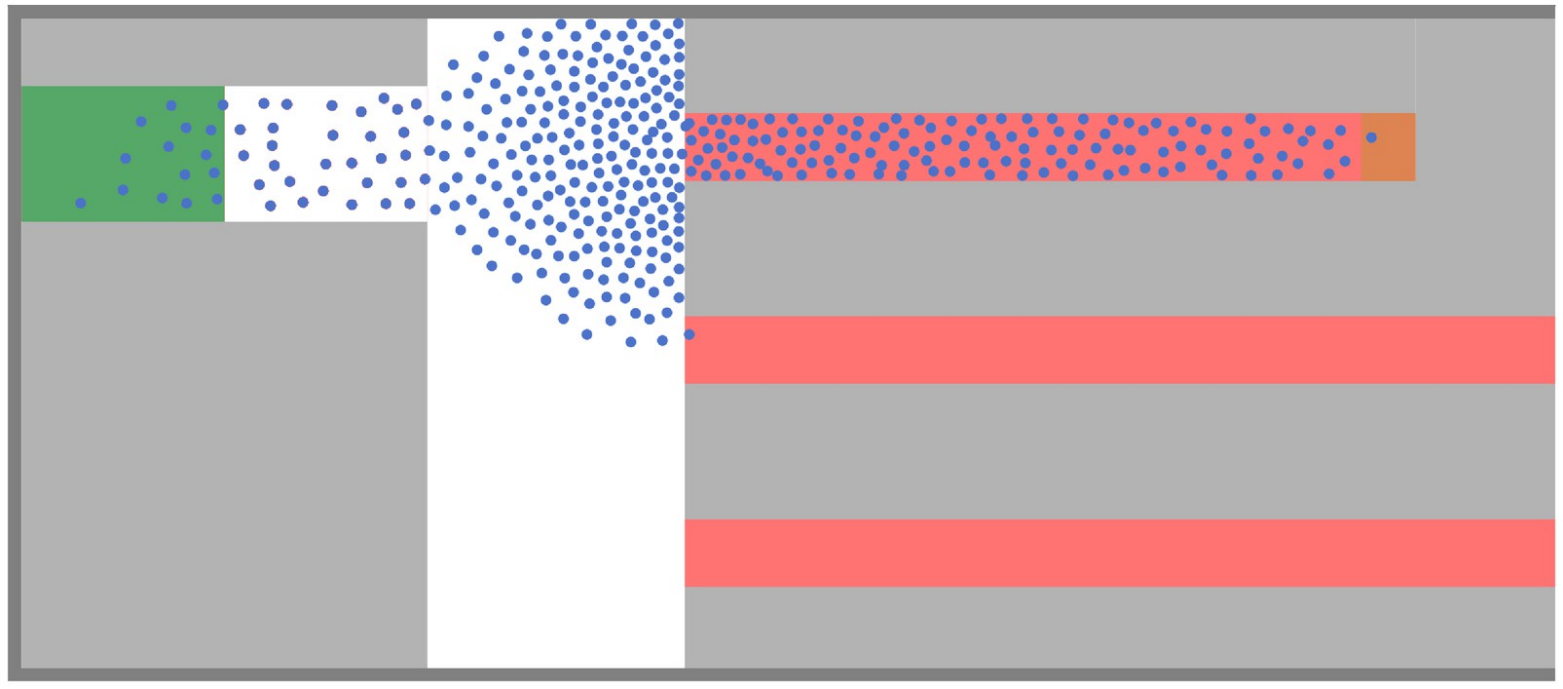
Setting without guidance. All agents take the short route (simulation time *t* = 250*s*). Bottleneck jamming occurs because the inflow is larger than the capacity of the short corridor.

**Fig 5:**
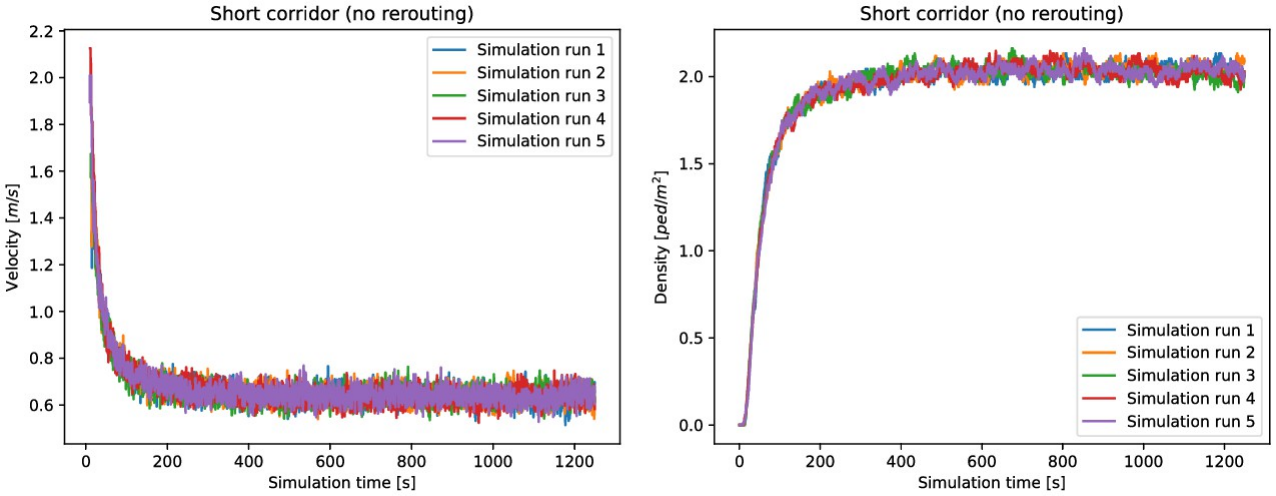
Velocity and density in the short corridor over the simulation time in the setting without guidance. A steady-state flow is reached at approximately 250*s*. Densities and velocities before 250*s* are not included in the evaluations. For each compliance rate, we repeat the simulation five times. Since the variance in the results is small this appears a good compromise between accuracy and computational effort.

When interpreting our simulation results, we first look at the case without guidance where all agents take the short route, see Fig 4. For the performance evaluation, we look at the steady state of the system, which is reached when densities and velocities stagnate. We observe that a steady-state flow has been reached after about 250*s*, see Fig 5. We exclude from the analysis all measurements before 250*s*.

Table 3 lists the measured values for the quantities of interest on the short route. For the density and the velocity, the median and the mean values are similar. However, the mean travel time is considerably lower than the median travel time. Since this indicates that the travel time does not follow a normal distribution we use the median travel time for further evaluations.

**Table 3:** Statistics of the short corridor for the setting without guidance. Mean values, standard deviation, median, and further statistical quantities for the density and the velocity are computed using 12500 samples. The median and the mean value are similar for the density and the velocity. To compute the statistics of the travel time, we evaluate the individual travel times of 14613 agents. The mean travel time is considerably lower than the median travel time, indicating that it does not follow a normal distribution.

Quantity of interest	mean	std	min	25%-quar.	med	75%-quar.	max	counts
Density [*ped*/*m*^2^]	2.03	0.04	1.87	2.00	2.03	2.06	2.16	12500
Velocity [*m*/*s*]	0.64	0.03	0.51	0.62	0.64	0.66	0.77	12500
Travel time [*s*]	195	84	70	147	178	216	898	14613

The route length is approximately 50*m*. With a mean free-flow velocity of 1.34*m*/*s*, the default parameter of the locomotion model, one would expect an average travel time of around 37*s*, if agents walked freely. Instead, we observe congestion in the short corridor at a mean density of 2.0*ped*/*m*^2^ and with a walking speed reduced to 0.65*m*/*s* on average. The density value of 2.0*ped*/*m*^2^ corresponds to E as a level of service. Moreover, the median travel time of 178*s* is almost five times longer than expected in a free flow regime, see Table 3. Furthermore, the jamming criterion is fulfilled. The capacity of the short corridor is between 1.3*ped*/(*ms*) and 1.54*ped*/(*ms*), see supporting information (S2 Appendix). This is lower than the inflow 1.60*ped*/(*ms*). Thus our results are plausible.

### A small proportion of compliant people suffice to avoid jamming

**Fig 6:**
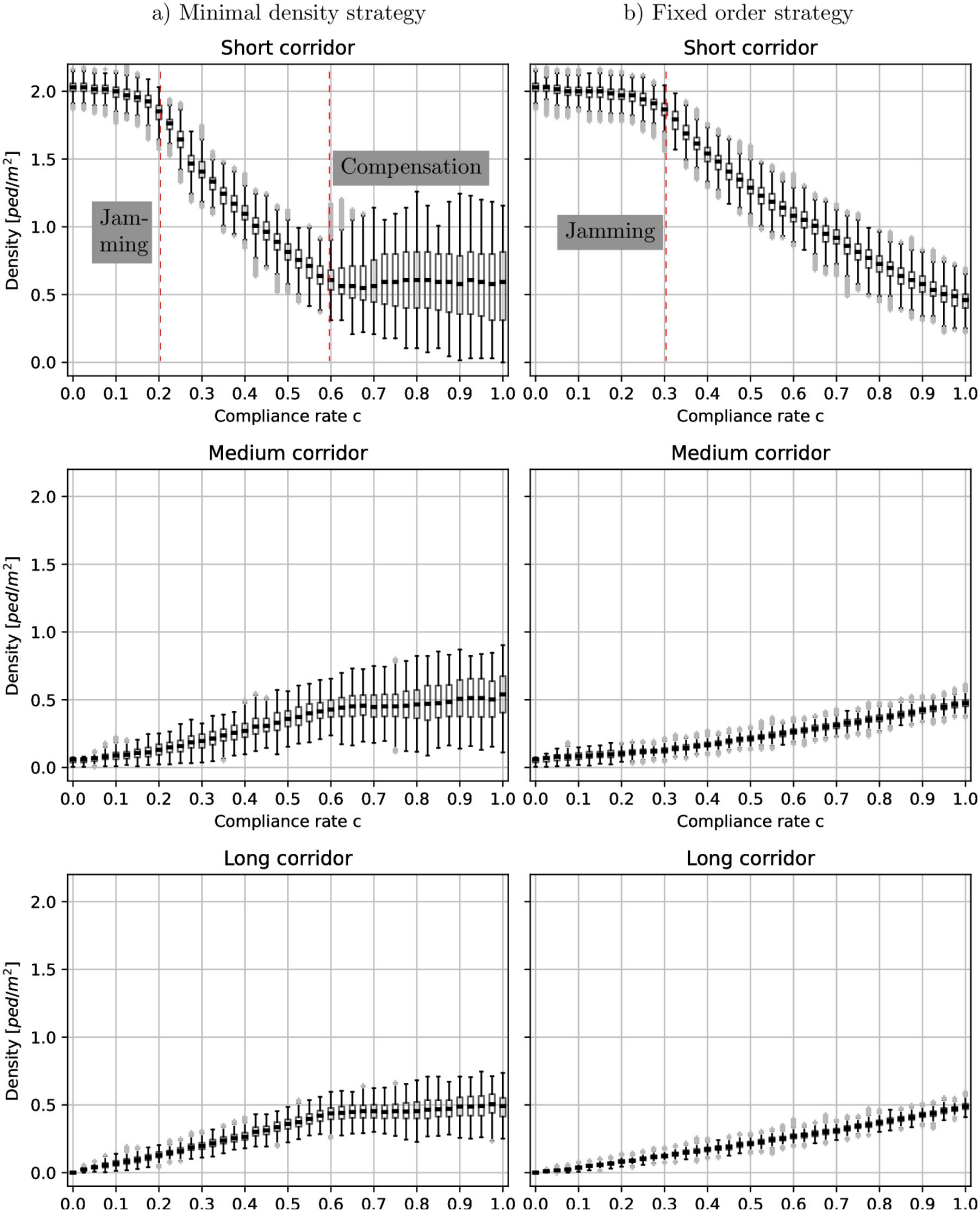
Boxplots of the densities per route. The boxes represent the 25% and 75%-quartiles. The thick black line in the box indicate the median value. Whiskers do not extend up and down from the box more than 1.5 times the interquartile range (75%-quartile—25%-quartile). Values outside the whiskers are considered as outliers (gray dots). We observe different states on the short route. If the compliance rate is small, jamming occurs. With better compliance, the density decreases. For a compliance rate above *c* > 0.6, the density is almost constant for the minimal density strategy. In contrast to the fixed order strategy, the minimum density strategy then fully compensates the lack of compliance.

**Fig 7:**
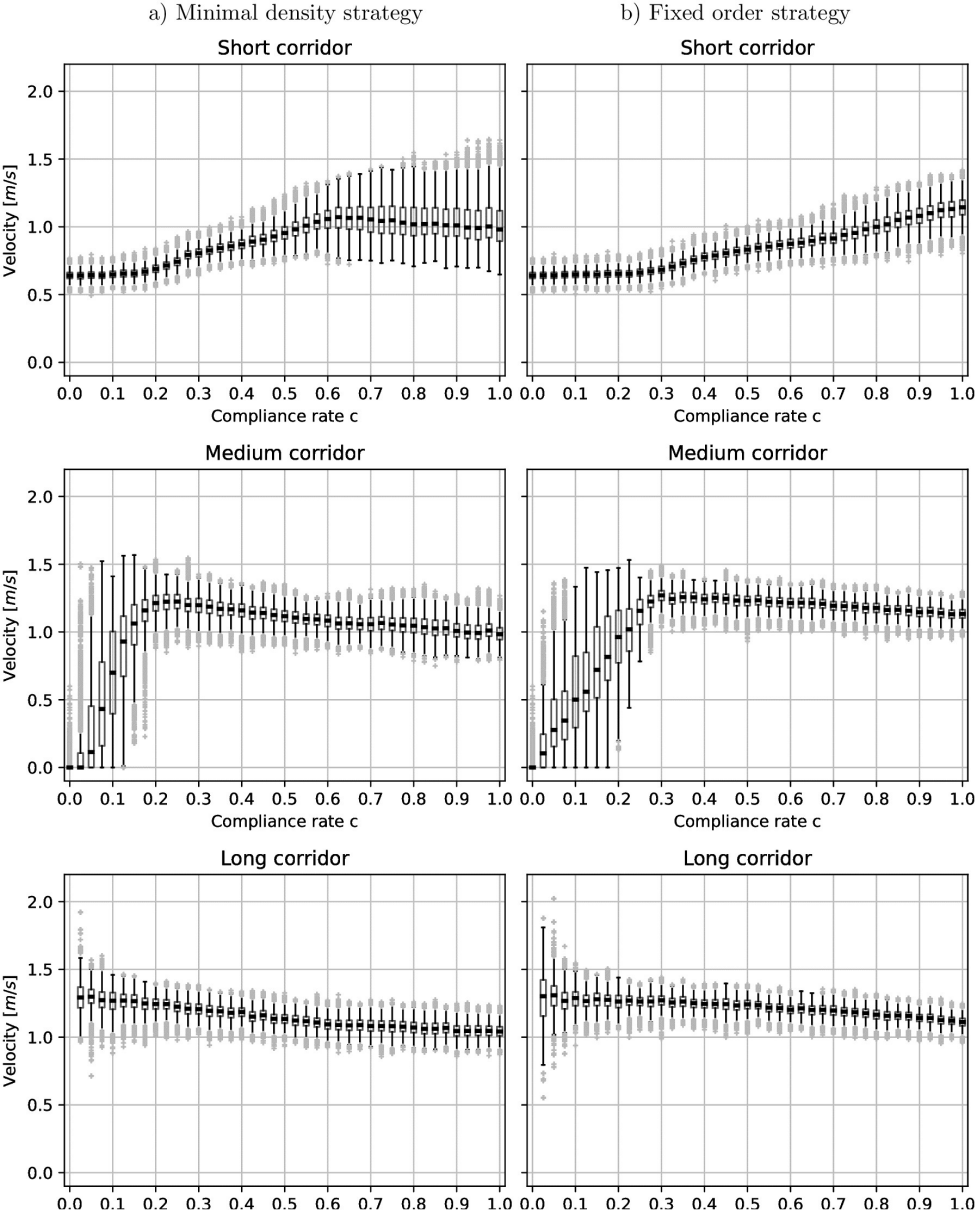
Boxplots of the velocities per route. The boxes represent the 25% and 75%-quartiles. The thick black line in the box indicates the median value. Whiskers do not extend up and down from the box more than 1.5 times the interquartile range (75%-quartile—25%-quartile). Values outside the whiskers are considered as outliers (gray dots). In the setting without guidance, or total lack of compliance, (*c* = 0), the velocities in the short corridor become very small. Note that, the velocities of *v* < 0.6*m*/*s* in the medium route are simulation artifacts caused by agents waiting to enter the short route in the middle corridor. This happens if the compliance rate is very low, and the entrance of the short route is jammed (see Fig 4).

Whether or not a guidance strategy avoids congestion depends on the compliance rate. If no one listens to the route recommendation (*c* = 0), the situation is just as if there were no guidance. With the jamming calculations in mind, we expect congestion, for the fixed order setting, if the compliance rate *c* < 0.28. The upper third of Fig 6 depicts the density over the compliance rate in the short corridor. We observe that the density increases when the compliance rate decreases. With compliance rates below about *c* = 0.25 the density stagnates. This is close to *c* = 0.28 we estimated through the jamming criterion. Consistently, the measured velocity also stagnates when the compliance rate drops below *c* = 0.25, see Fig 7.

**Fig 8:**
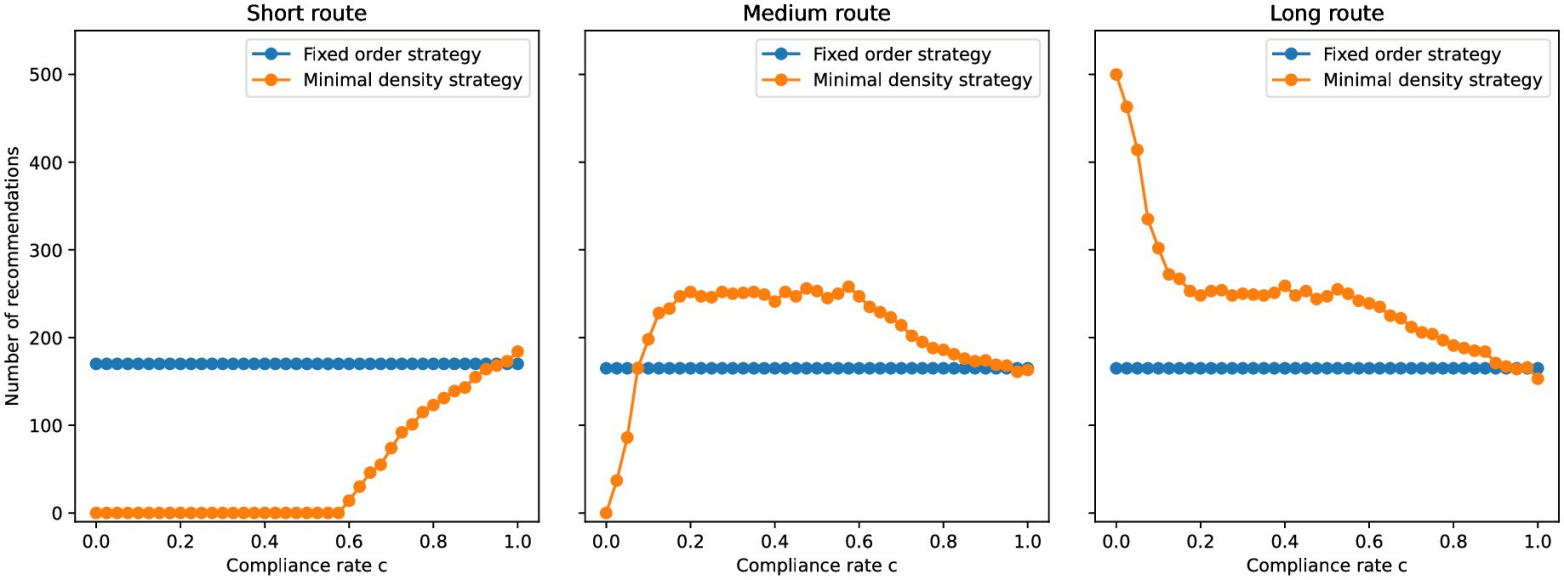
Number of route recommendations per corridor in dependency of the compliance rate. For the fixed order strategy, by definition, the number of recommendations does not change. The minimum density strategy recommends the short route only for high compliance rates *c* > 0.6. When the compliance rate drops very low, *c* < 0.15, it exclusively suggests the long route.

**Fig 9:**
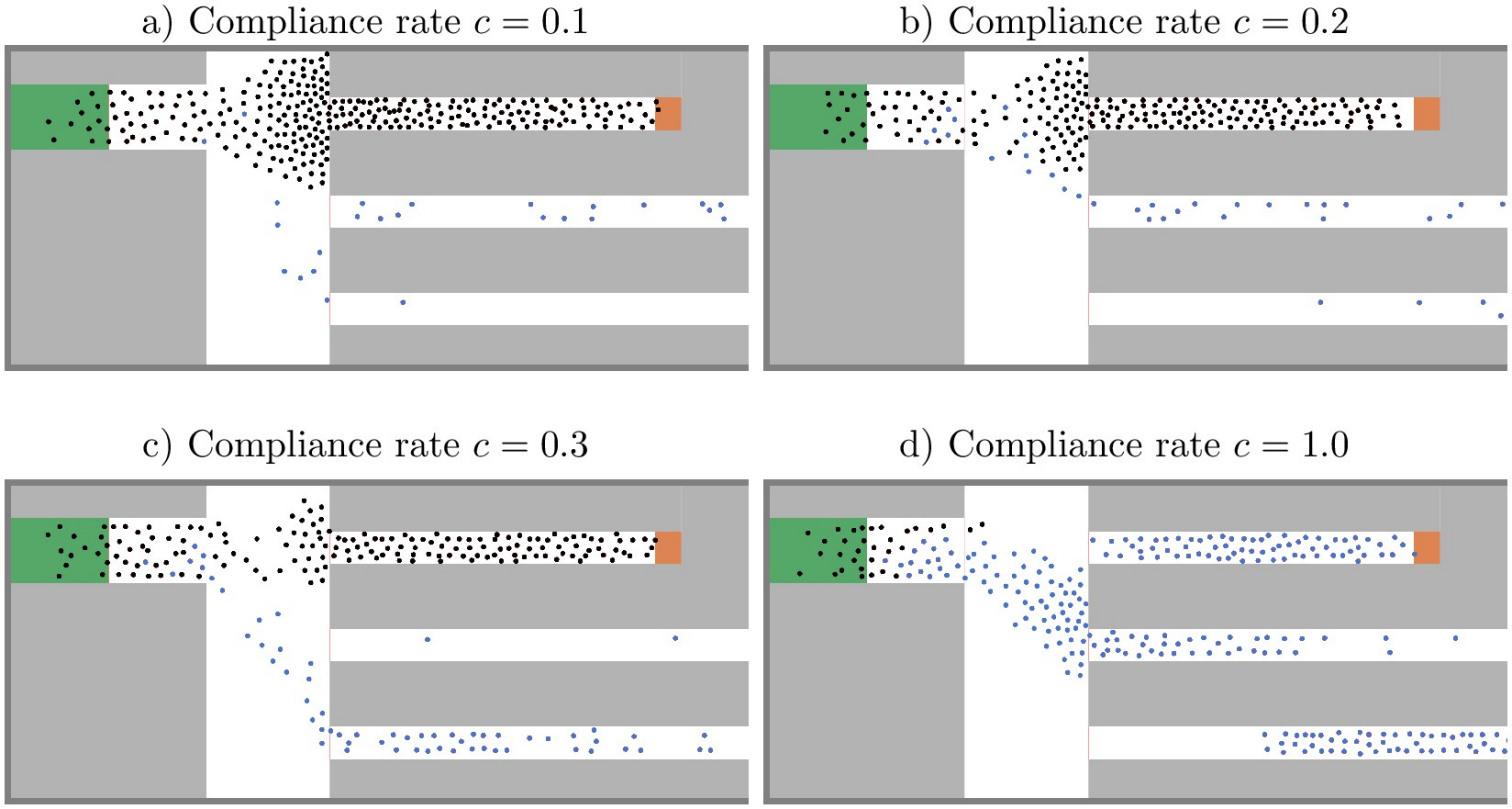
Minimal density strategy at simulation time *t* = 250*s*. a) and b) The congestion in front of the bottleneck dissolves with increasing compliance rate; d) At *c* = 0.3, it has almost completely disappeared. Only non-compliant agents (black) remain in the short corridor where the density is high. Compliant agents (blue) enjoy a better level of service.

For the minimum density strategy, we observe a similar effect on the densities when the compliance rate is only about *c* ≈ 0.2, see Fig 6. Thus, compared to the fixed-order strategy, a lower compliance rate suffices to avoid congestion. The minimum density strategy no longer recommends the short corridor at all if the compliance rate drops below *c* < 0.6, see Fig 8. Then, the portion of people that are directed away from the short route is *q* = 1, which we can put into Eq 3 to estimate a minimal compliance rate to avoid congestion. We get *c* = 0.19, which is in line with our observations, see Fig 9. The lower the compliance rate, the more non-compliant agents congregate in the short corridor so that the guidance strategy favors the other, emptier, corridors. With this, the minimum density strategy fully compensates the lack of compliance in our scenario for compliance rates of *c* = 0.6 and above. Between compliance rates of 0.2 and 0.6 it outperforms the fixed order strategy.

### Both guiding strategies reduce the travel time

**Fig 10:**
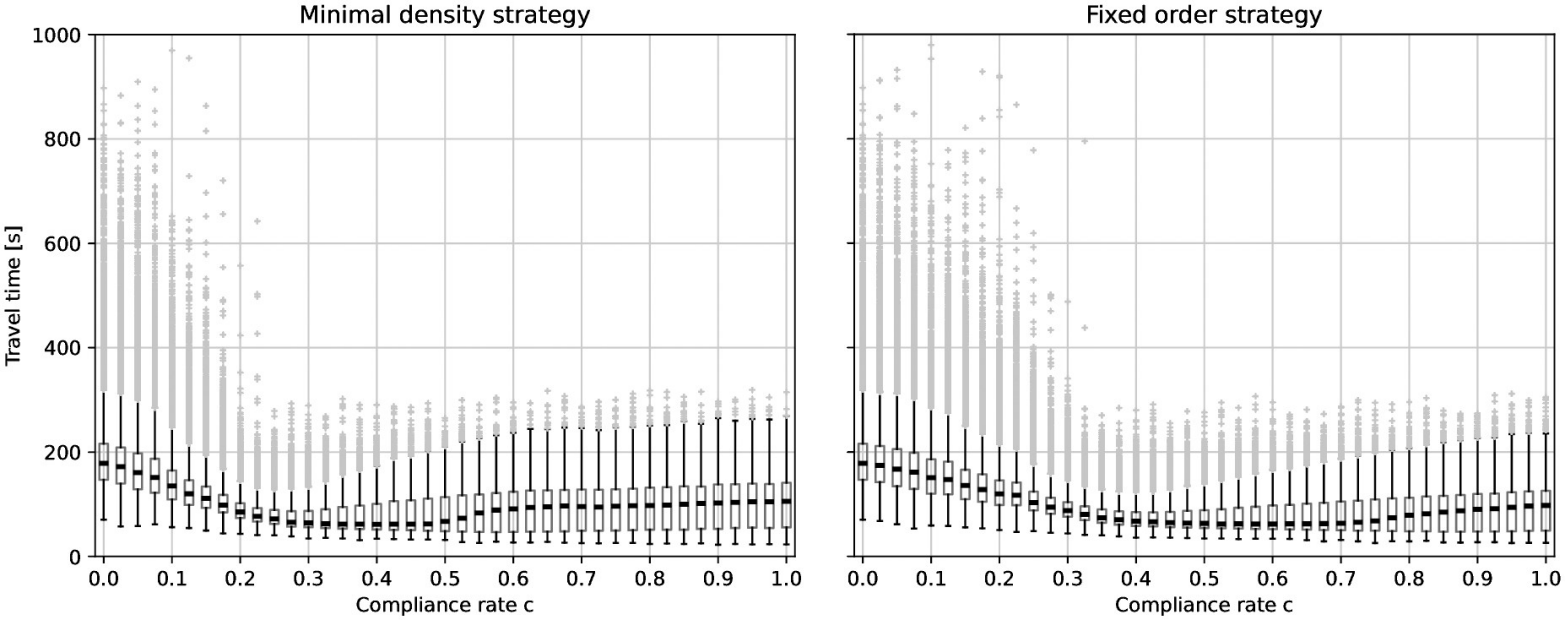
Boxplots of the travel time. The boxes represent the 25% and 75%-quartiles. The thick black line in the box indicates the median value. Whiskers do not extend up and down from the box more than 1.5 times the interquartile range (75%-quartile—25%-quartile). Values outside the whiskers are considered as outliers (gray dots). Left: for the minimal density strategy, the individual travel times are high when the compliance rate is very low. At a compliance rate of about *c* = 0.2, the variance drops, and outliers are no longer extreme. Right: for the fixed order strategy, we observe a similar effect, but at a higher compliance rate of about *c* = 0.3.

**Fig 11:**
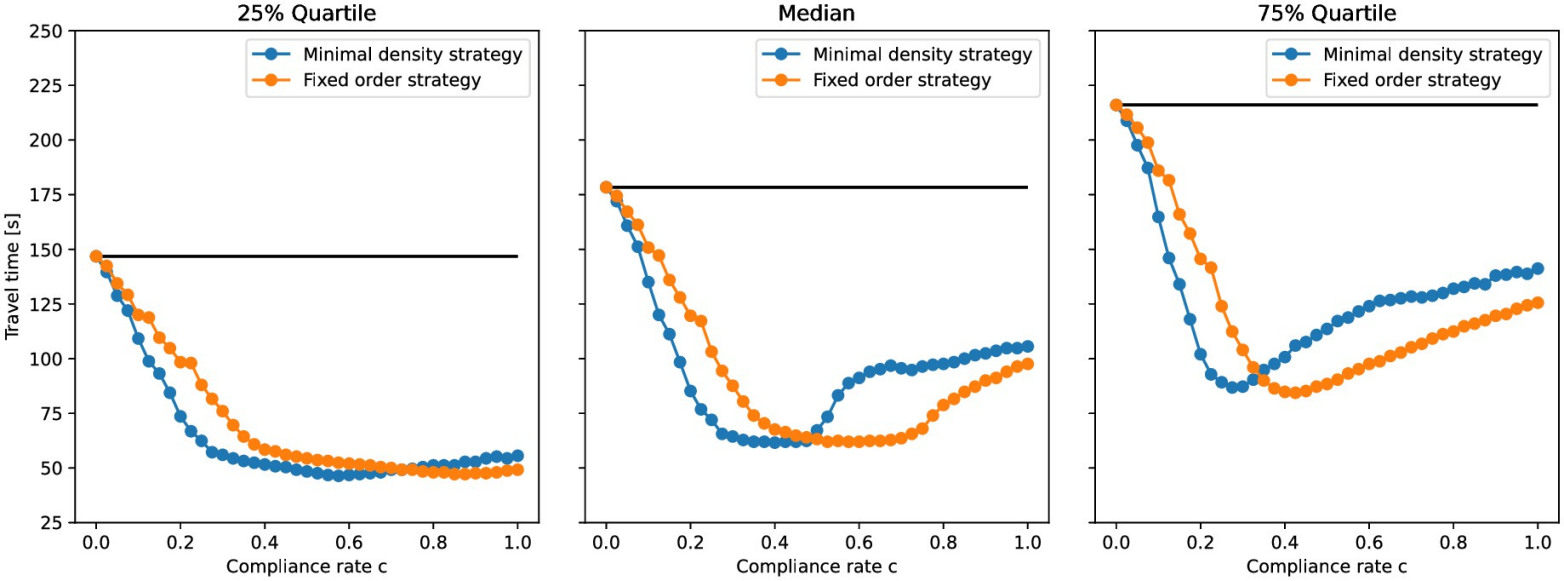
25%,50% and 75%-quartiles of the travel time in dependency on the compliance rate. The black line represents the setting without guidance, that is, a compliance rate of *c* = 0. The orange line represents the fixed order strategy and the blue line the minimal density strategy. Both guiding strategies reduce the travel times so that, for any compliance rate, the quartiles are always below the line that shows the travel time quartiles without guidance.

We have seen, that without guidance there is congestion resulting in high travel times in our scenario. In Fig 10, which shows the box plots of travel times in dependency of the compliance rates, one can observe the effect of guidance: As long as the compliance rate is low there is a high variance in the individual travel times. Some outliers even have very long travel times. For the minimal density strategy, this changes at a compliance rate of *c* ≈ 0.2. The variance drops, and outliers are no longer extreme. For the fixed order strategy, this effect occurs later, at *c* ≈ 0.3. Note that, as before, these are the minimum compliance rates to avoid congestion that we estimated using the jamming criterion. In the next step, we look at the 25%, 50% and 75% quartiles of the travel time, see Fig 11. The black lines refer to the setting without guidance (*c* = 0) and represent an upper limit. The graphs are always below the black line, thus, both guiding strategies reduce the travel times in our scenario. Interestingly, the median travel time is not minimal when everybody follows instructions (*c* = 1.0).

In Fig 11 (middle), we observe that the median travel time is around 100*s* for *c* = 1.0. The minimal median travel time (60*s*) is achieved when the compliance rate is *c* ∈ [0.2, 0.6] for the minimal density strategy and *c* ∈ [0.3, 0.75] for the fixed order strategy. The lower bounds of the intervals, that is 0.2 and 0.3 respectively, are equal to the compliance rates for which we found that congestion is resolved. Hence, we find that in our scenario, the median travel time becomes minimal as soon as congestion is resolved. While this finding may be scenario-specific, that is, not applicable in general, we found it curious and would be interested to learn whether it carries over to other scenario types.

It is easier to explain why the minimum travel time is at an intermediate compliance level and that, while full compliance evens out densities, it does not optimize travel times. Imagine a compliance rate of *c* = 0.3. In this case, at least 70% (70% = 1 − *c*) of the agents take the short corridor. Thanks to sufficiently many other agents taking longer routes, there may be elevated density on the short path, but no congestion. This means short travel times for most. On the other hand, if 100% of the agents reject the route recommendation, the setting is equivalent to the scenario without guidance where the travel time peaks, see Fig 11.

**Fig 12:**
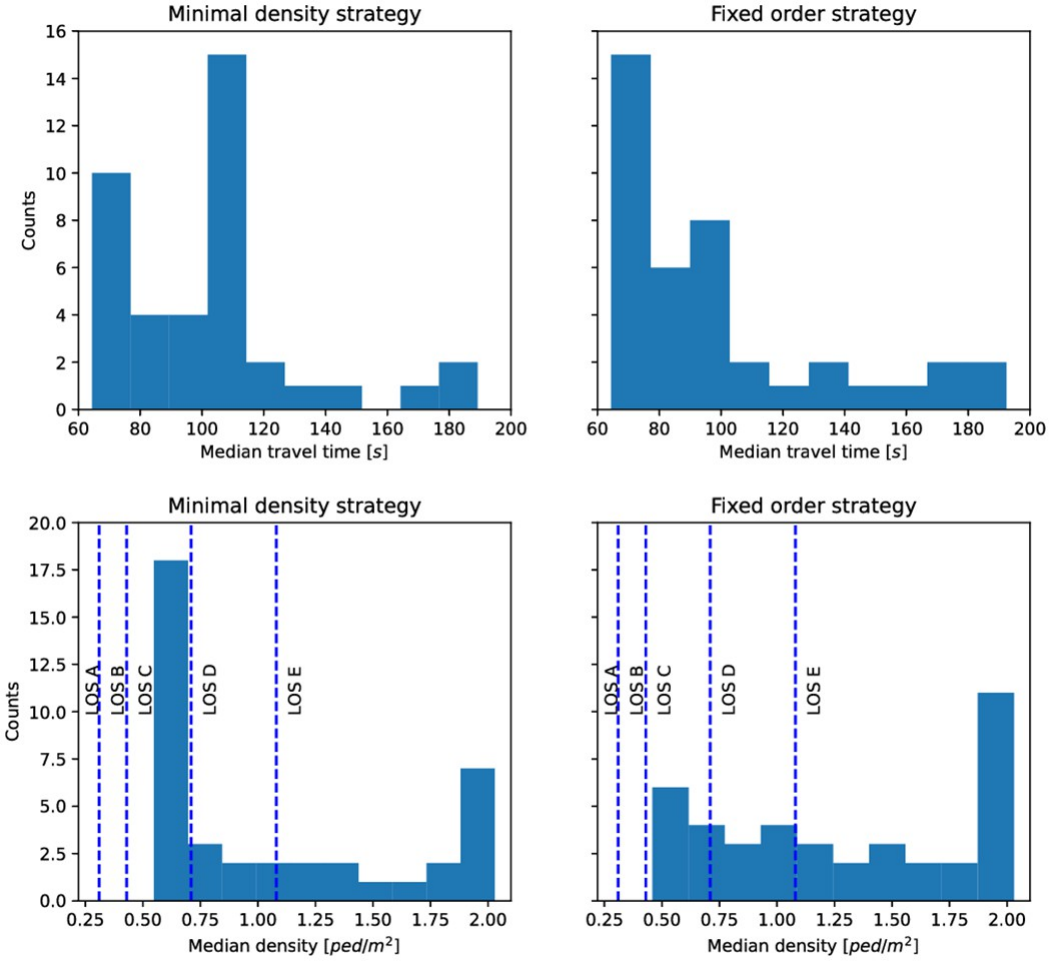
Comparison of distributions. Top: Distributions of the median travel time for positive compliance rates, *c* > 0, from Fig 10. The sample size is 40 for each strategy. Statistical tests do not reject the hypothesis that the distributions are equal. We assume that there is no difference. Bottom: We compare the median density values for positive compliance rates, *c* > 0, measured in the short corridor from Fig 6. The sample size is 40 for each strategy. The density distributions, and thus, the level of service (LOS), differ statistically.

We would like to know which of the two strategies yields lower median travel times, see Fig 12. To be able to select a suitable statistical test, we test the median travel time distributions for normality by employing KS-tests. We get *p* = 0.000 for both strategies and, thus, reject the null hypothesis that the distributions are normally distributed.

We then compare the two distributions of the median travel time with a Mann-Whitney-U-test. Here, the null hypothesis is that for random samples X and Y from the two populations, the probability of X being greater than Y is equal to the probability of Y being greater than X. That means we test for similarity. With *p* = 0.085, we accept the null hypothesis which means that statistically, the median travel time distributions of the two guiding strategies are equal.

### Better level of service with the minimal density strategy

In the following, we use the mean densities to compare the performance of the two strategies. With the minimum density strategy, the densities in the three corridors are approximately equal if at least 60% of the people follow instructions. Then, this strategy can fully compensate the lack of compliance. For *c* < 0.6, only the medium and long routes are recommended, see Fig 8. With the fixed order strategy, on the other hand, densities only coincide for full compliance (*c* = 1). For all lower compliance rates, *c* < 1, the density in the short corridor surpasses that in the other corridors. In other words, the strategy cannot compensate for the lack of compliance.

With this in mind, we would like to know which of the two strategies offers a better level of service in the short corridor, and proceed to compare the median densities, see Fig 12. KS-tests reveal, with *p* = 0.00 for both strategies that the median densities are not normally distributed. As before we use a Mann-Whitney-U-test to compare the distributions of the median densities, with the null hypothesis that, for random samples X and Y from the two populations, the probability of X being greater than Y is equal to the probability of Y being greater than X. The alternative is that one distribution is stochastically greater than the other, which implies different distributions. This time, with *p* = 0.031, we reject the null hypothesis and conclude that the density distributions for the two guidance strategies differ statistically. Indeed, the median value of the density for the minimal density strategy corresponds to a level of service D, while we only attain a level of service E for the fixed order strategy.

## Discussion

### Summary

We looked at an abstracted model of a subway station where congestion is a recurring problem with high traffic volumes, causing long travel times and high densities. We investigated two guiding strategies based on heuristics that aim to distribute the crowd over different routes: The first strategy alternated which routes it recommends to the crowd in a fixed order, while the second suggested the route with the lowest density. Our goal was to find out how suitable each strategy is to reduce densities, that is, to improve the level of service, and, lower travel times, if only part of the crowd follows instructions. Crucially, we gave up the assumption of full compliance that many control systems make.

For our simulation studies, we stripped the topography of detail and reduced it to its key characteristics. This left us with three corridors of different lengths that agents reach from a large hall. All agents received the same information, at a fixed interval, thus avoiding the problem of conflicting directions within a group. We estimated minimal compliance rates to resolve congestion in front of the corridors, using the established ‘jamming criterion’. This allowed us to check our simulation results for plausibility. We ran simulations, varying compliance rates, to investigate the performance of the two strategies.

We found that both strategies, despite their simplicity, shorten the travel time and improve the level of service. The minimal density strategy consistently outperformed the fixed order strategy. While both strategies enjoyed equal success with the travel times, the minimal density strategy, which responds to the system’s current state, always offers a better the level of service. This also seems fair, since it rewards compliance, in this case, the willingness to take a longer path, with a good level of service. Non-compliant people, on the other hand, risk long travel times in case of jamming. In the absence of congestion, they will reach their destination quickly, but they will have to accept a low level of service. We found that even at low compliance rates, the minimal density strategy can fully resolve congestion, which in turn reduces the overall travel time. In our scenario, it is sufficient if only every fifth person follows instructions. This corresponds to a compliance rate of 20% which, we think, is within the realm of possibility in real life.

The fixed order strategy recommends the short corridor regardless of its utilization. Compliant and non-compliant agents will mingle in this area. Persons who selected this path following instructions may well doubt the usefulness of the guidance systems if they face congestion—and ignore the system next time. This seems undesirable.

### Limitations and open research questions

In our study we dropped the assumption of full compliance, which has often been made implicitly before. However, with the goal to make initial progress, we strongly simplified several points, the influence of which we think should be investigated in future:

We assume that the compliance rate to be uniformly distributed and to be the same in the whole area of investigation. Also, agents accept or reject the route recommendation without delay, which means that we ignore reaction times.

We are also aware, that the estimated minimum compliance rates are scenario-specific, clearly depending on the capacity, which in turn depends on the topography, and also on the inflow. Our studies fixed both, capacity and inflow, so that our quantitative results do not generalize. We do believe, however, that the qualitative results hold and, also, that the approach can be transferred.

Another issue is the scheduling of recommendation updates. Our agents all received the same information every 10*s*. Other time intervals should be considered. In the case of the minimal density strategy, one might even give up the information scheduling but should keep in mind that conflicting instructions in a group and between groups may occur if the density fluctuates. This problem must be tackled because groups will not separate. Conflicting instructions can also come from a change in recommendation after 10*s*. We resolved this, somewhat artificially, by letting the agents only respond to the first route recommendation. A real system would have to be engineered to avoid conflicts.

With our focus on understanding fundamental dependencies, we looked at an extremely abstracted scenario where all corridors were straight and had the same width. In reality, bends and varying widths, stairs, and bad surface conditions create bottlenecks within the routes, affecting travel time and density. It would be interesting to see, how the minimum density strategy performs in more complex real-world scenarios.

Finally, there are psychological aspects that we could not address within the scope of this study. Firstly, we only considered individuals. Introducing small groups, like families, who stick together, as we did in several trial runs, did not much alter the simulation results. However, there are social groups within a crowd, who adhere to common norms and exhibit similar behavior. If one thinks of soccer fans at an underground station, this might have a significant influence.

We designed our contribution as a proof of concept, wanting to find out, whether guiding strategies would work in principle. As a consequence, we largely ignored the implementation issues of a real system. One such issue is, how to measure densities, be it from video footage or new generation mobile phone signals. Another, very big open question is, how to inform people about the measures. We were content with mentioning dynamic arrows or apps. Luckily, a lot of research is being conducted in both areas.

## Conclusion

The goal of this study was to assess whether simple guiding strategies, based on heuristics instead of complex and, probably, sensitive feedback loops, are suitable to redirect crowds. In particular, we wanted to investigate performance when facing low compliance with instructions. We proposed a strategy that alternates route suggestions, irrespective of utilization, and a strategy that responds to the current state by suggesting the path with the lowest crowd density. We investigated both through simulation studies. Both strategies significantly improved the situation, lowering travel times. The minimal density strategy offered a better level of service. Our findings demonstrate, that simple guiding strategies based on heuristics are indeed able to achieve an acceptable performance. We argue that they should be realized in preference to elaborate systems that depend too much on exact measurements and may not be able to cope with low compliance. Indeed, we think that the minimal density strategy is a good candidate for the first implementation in a real system.

In view of this, we consider our contribution as the first step towards an emerging technology. The minimal density algorithm could be used to automatically generate route recommendations in dynamic lighting guidance systems or apps. Yet, many open questions remain. We would like to draw the readers’ attention to some points that we think are especially important: We know very little about the compliance rate and assumed it to be uniformly distributed and independent of the location at which people find themselves. To gain more insight we intend to conduct a survey that makes use of our underground scenario. This necessitates a cross-disciplinary project with psychologists. We also plan to build a more faithful model of the underground topography, where stairs and bottlenecks en route are no longer ignored. Also, we plan to evaluate the performance of the strategies using cost functions.

## Supporting information

S1 Appendix Relationship between minimum density strategy and on-off controller. (PDF)

S2 Appendix Capacity estimation. (PDF)

S3 Appendix Locomotion model: Fixed parameter values. (PDF)
