# Supplementary material for: Guiding crowds when facing limited compliance: Simulating strategies
Source: PLoS One. 2022 Nov 11;17(11):e0276229. doi: 10.1371/journal.pone.0276229 (PMC9651580; doi:10.1371/journal.pone.0276229)
Supplement: S1 Appendix — (PDF) [file pone.0276229.s001.pdf]

S1 Appendix. Relationship between minimum density strategy and on-off controller.

The minimal density strategy is a simple heuristic that recommends the route where the density is the lowest. However, it can also be represented as a system of on-off controllers (also known as bang-bang-controllers or two-step-controllers). We think this relationship might be interesting for our readers, because some proposed guiding strategies [1, 2] are based on-off controllers. In contrast to the proposed systems, we use a dynamic desired value and we allow only one controller set active at a time.

Let ρ_k^i be the density in corridor i at time step (k) with i $\in N$ [1]. ρ_{ref} is the desired density (reference density). We define the measurement error according to [3] as

$$e_k^i = \rho_k^i - \rho_{ref}^i \tag{1}$$

According to [3], the control action u_k^i at time k for route i is defined as

$$u_k^i = \begin{cases} 1 & e_{k-1}^i = 0 \\ 0 & e_{k-1}^i > 0 \end{cases} \tag{2}$$

$u_k^i = 0$ means that no person should use exit or corridor i. $u_k^i = 1$ means that the exit or corridor is open.

Let $D = [1, 2, .., n]$, where n is the number of routes and the routes are order according to their length (1: shortest, ..., n: longest route). For each route (i), there is one on-off-controller that decides whether a certain route (i) should be recommended or not. We would like to avoid conflicting information, that is, recommending several corridors at a time. We recommend corridor m:

$$m = \min(\arg\min_{i \in D} u^i(\rho_{ref})) \tag{3}$$

If there are multiple routes recommended, the outer min condition makes sure that the shorter route is recommended. To distribute people over the corridors, we choose a dynamic reference density $\rho_{ref,k}$ that is defined as

$$\rho_{ref,k} = \min \rho_{i,k-1} \tag{4}$$

Since the measurement error e_k is only $e_k = 0$ for the corridor(s) where the density is minimal, the expression (Eq. 3) simplifies to:

$$m = \min(\arg\min_{i \in D} \rho_{i,k-1}) \tag{5}$$

This is exactly how we defined the minimal density strategy. Note that for u in Eq. 3 any control algorithm is conceivable from a theoretical perspective.

References

1. H. Ren, Y. Yan, and F. Gao. Variable guiding strategies in multi-exits evacuation: Pursuing balanced pedestrian densities. *Applied Mathematics and Computation*, 397, 2021.

2. Jie Xu, Yao Ning, Heng Wei, Wei Xie, Jianyuan Guo, Limin Jia, and Yong Qin. Route choice in subway station during morning peak hours: A case of guangzhou subway. *Discrete Dynamics in Nature and Society*, 2015:1–8, 03 2015.

3. Xiangxia Ren, Jun Zhang, Shuchao Cao, and Weiguo Song. Experimental study on elderly pedestrians passing through bottlenecks. *Journal of Statistical Mechanics: Theory and Experiment*, 2019(12):123204, dec 2019.
