# Supplementary material for: Guiding crowds when facing limited compliance: Simulating strategies
Source: PLoS One. 2022 Nov 11;17(11):e0276229. doi: 10.1371/journal.pone.0276229 (PMC9651580; doi:10.1371/journal.pone.0276229)
Supplement: S2 Appendix — (PDF) [file pone.0276229.s002.pdf]

S2 Appendix. Capacity estimation.

We want to know which compliance rate is necessary to avoid congestion. For that purpose, we need the inflow and the capacity of the route in which congestion occurs. The inflow in this study is $1.6ped/(ms)$. We measure the density and velocity in the unguided setting. We use these values to estimate the capacity.

Figure A depicts the flow-density relation and the velocity-density relation. The specific flow is computed using $J_S = \rho v$. The maximum flow is $J_C = 1.58ped/(ms)$. This value is higher than values from handbooks: Weidmann [1] proposes a value of $1.23ped/(ms)$, Fruin [2] proposes $1.43ped/(ms)$, while the SFPE handbook [3] provides a value of $1.30ped/(ms)$. We think that our peak value of $1.58ped/(ms)$ is caused by the measurement method. We measure the velocities and densities every $0.4s$. The flow values would be lower if we used a larger time interval. In order not to overestimate the capacity, we use a polynomial regression model to find an average value. The maximum value computed with the regression model is $J_C = 1.30ped/(ms)$. This value is in line with the handbook values.

Figure A. Fundamental diagrams for the short route. The specific flow is $J_S = \rho v$. The maximum flow is $J_C = 1.58ped/(ms)$.

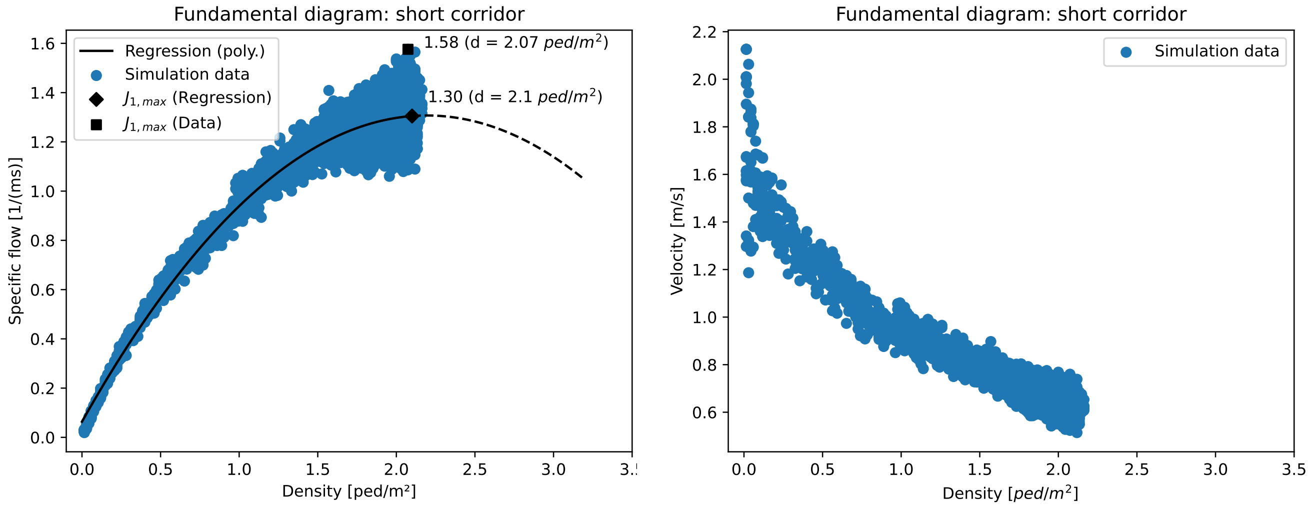

References

1. Ulrich Weidmann. *Der Fahrgastwechsel im öffentlichen Personenverkehr.* PhD thesis, Eidgenössische Technische Hochschule Zürich, 1994. Dissertation ETH Nr.10630.

2. John J. Fruin. *Pedestrian Planning and Design.* Metropolitan Association of Urban Designers and Environmental Planners, New York, 1964.

3. Morgan J. Hurley, Daniel T. Gottuk, John R. Jr. Hall, Kazunori Harada, Erica D. Kuligowski, Milosh Puchovsky, Jose L. Torero, John M. Jr. Watts, and Christopher J. Wieczorek. *SFPE Handbook of Fire Protection Engineering.* Springer, New York, 5 edition, 2016.
