# Supplementary material for: Guiding crowds when facing limited compliance: Simulating strategies
Source: PLoS One. 2022 Nov 11;17(11):e0276229. doi: 10.1371/journal.pone.0276229 (PMC9651580; doi:10.1371/journal.pone.0276229)
Supplement: S3 Appendix — (PDF) [file pone.0276229.s003.pdf]

S3 Appendix. Locomotion model: fixed parameter values.

We use the Optimal Steps Model [1] to simulate the locomotion of the crowd. The following tables contain the values for the model parameters we used in this study. They correspond to the default values.

Table A. Parameter values of the Optimal Steps Model. For a detailed description of each parameter, see `AttributesOSM` in the implementation [1].

Parameter	Value
`stepCircleResolution`	4
`numberOfCircles`	1
`optimizationType`	"NELDER_MEAD"
`varyStepDirection`	true
`movementType`	"ARBITRARY"
`stepLengthIntercept`	0.4625
`stepLengthSlopeSpeed`	0.2345
`stepLengthSD`	0.036
`movementThreshold`	0.0
`minStepLength`	0.1
`minimumStepLength`	true
`maxStepDuration`	1.7976931348623157E308
`dynamicStepLength`	true
`updateType`	"EVENT_DRIVEN"
`seeSmallWalls`	false

Table B. Parameter values of the potential functions used in the Optimal Steps Model. For a detailed description of each parameter, see `AttributesPotentialCompactSoftshell` in the implantation [1].

Parameter	Value
`pedPotentialIntimateSpaceWidth`	0.45
`pedPotentialPersonalSpaceWidth`	1.2
`pedPotentialHeight`	500
`obstPotentialWidth`	0.8
`obstPotentialHeight`	6.0
`intimateSpaceFactor`	1.2
`personalSpacePower`	1
`intimateSpacePower`	1

Table C. Parameter values of floor field used in the Optimal Steps Model. For a detailed description of each parameter, see `AttributesFloorField` in the implementation [1].

Parameter	Value
`createMethod`	"HIGH_ACCURACY_FAST_MARCHING"
`potentialFieldResolution`	0.1
`obstacleGridPenalty`	0.1
`targetAttractionStrength`	1.0
`standardDeviation`	0.7
`type`	"UNIT"
`obstacleDensityWeight`	3.5
`pedestrianSameTargetDensityWeight`	3.5
`pedestrianOtherTargetDensityWeight`	3.5
`pedestrianWeight`	30.0
`queueWidthLoading`	1.0
`pedestrianDynamicWeight`	6.0
`loadingType`	"CONSTANT"
`width`	0.2
`height`	1.0

References

1. Benedikt Kleinmeier, Benedikt Zönnchen, Marion Gödel, and Gerta Köster. Vadere: An open-source simulation framework to promote interdisciplinary understanding. *Collective Dynamics*, 4, 2019.
